# Microvascular Genesis of Diseases: From Hypothesis to Theory

**DOI:** 10.3390/life16020314

**Published:** 2026-02-11

**Authors:** Ruslan A. Nasyrov, Veronika A. Galichina, Anna O. Drobintseva, Daria V. Tonkonog, Elena Yu. Kalinina, Alexandra A. Agafonnikova

**Affiliations:** 1 Department of Pathological Anatomy, St. Petersburg State Pediatric Medical University, Ministry of Health of Russia, St. Petersburg 194100, Russia; rrmd99@mail.ru (R.A.N.);; 2 Department of Histology and Embryology, St. Petersburg State Pediatric Medical University, Ministry of Health of Russia, St. Petersburg 194100, Russia; ao.drobintseva@gpmu.org; 3 Department of Human Anatomy, St. Petersburg State Pediatric Medical University, Ministry of Health of Russia, St. Petersburg 194100, Russia

**Keywords:** microvessels, genesis, histohematic barriers, cell, pathogen, immunohistochemistry

## Abstract

Despite progress in understanding the molecular mechanisms of diseases, the dominant paradigm in explaining pathogenesis remains the concept of a pathogen’s direct damaging effect on parenchymal cells. Based on years of research, the authors of this article propose a revision of traditional views on disease pathogenesis. We emphasize the pivotal role of the microvasculature. Existing morphological studies provide insufficient insight into the role of these structures in the development of the pathological process. We conducted a search in international databases to find literary sources current as of December 2025. As an evidence base for the presented concept, we used the results of our own studies published from 1989 to the present. Data from the literature on non-infectious diseases are also separately presented. Our novel data from investigation of infectious and non-infectious diseases demonstrate that even in the initial stages of a pathological process, the microvessels of organs become the primary target of damage. The cascade of pronounced changes in parenchymal cells triggered by this initial event determines the development of the disease. The work examines the cellular and molecular aspects of the interaction between microvessels, pathogens, and the surrounding tissue. The proposed concept provides an objective and fundamentally new explanation for known facts. An important contribution of this concept is its potential to reveal promising directions for further research and for developing innovative approaches to disease therapy.

## 1. Introduction

**Figure A1:**
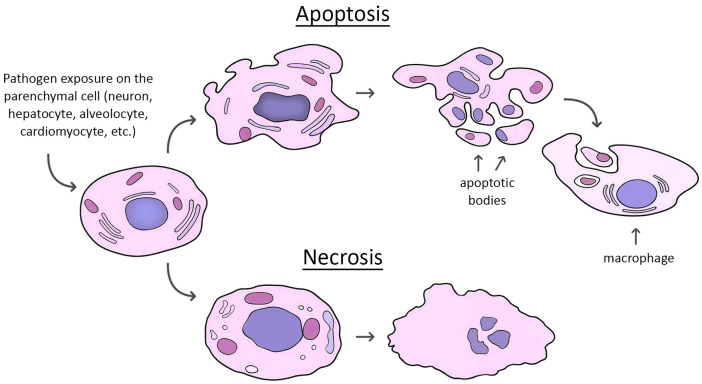
Schematic of direct pathogen exposure on the parenchymal cell according to the literature data.

Modern medical science is becoming more and more multidisciplinary, combining a wide variety of research areas. Despite discoveries in the field of multiomics [1] and the progress in the understanding of molecular mechanisms of diseases and the fundamental principles of pathology founded by R. Virchow [2], his ideas about the importance of histological examination and the cellular nature of diseases remain fundamental. At the same time, the dominant opinion explaining the pathogenesis of diseases remains the concept of the direct effect of pathogenic factors on cells. The hypothesis of the direct effect of pathogenic factors on cells suggests that damaging agents interact directly with various cell structures, disrupting their integrity, metabolism, and functions. This leads to an imbalance in intracellular homeostasis, which can be reversible or irreversible, up to cell death (Figure A1). Direct cytopathic/toxic effects can occur when infectious agents affect cells [3,4,5,6,7], explaining the pathogenesis of infectious diseases. It is important to note that the application of the mechanism of direct pathogenic action is not limited exclusively to infectious factors. It remains relevant for analyzing the influence of other exogenous and endogenous factors on the parenchymal cells of the body [8,9,10,11]. Meanwhile, the authors note difficulties in interpreting, for example, the direct effects of drugs on T cells in rheumatoid arthritis [12,13], the effects of insulin on beta cells [14], and ethanol on receptors [15].

The objective of the review is to summarize the results of morphological and clinical studies of microvessels, to identify patterns underlying the pathogenesis of infectious and non-infectious diseases, and to substantiate the microvascular genesis of diseases.

The authors of the article propose a revision of traditional views on the pathogenesis of infectious and non-infectious disorders based on long-term studies of various diseases, focusing on the key role of the vessels of the microcirculatory system (MCR) and their interaction with pathogens and the surrounding tissue. Morphological investigation of these structures and ideas about their role in the development of the pathological process are very fragmentary [16,17]. The humoral theory of C. Rokitansky is acquiring a new understanding in a broad sense [18]. The bloodstream delivers not only oxygen and the elements necessary for cell life, but also various pathogens. The microcirculatory bed is a complex network of the smallest vessels (arterioles, capillaries, and venules) and is critical for maintaining tissue homeostasis. Recent research in microcirculation has seen substantial progress, largely due to the introduction of technologies such as sublingual microscopy. Nevertheless, the implementation of these scientific findings in a clinical practice presents difficulties, as noted in the review [19]. A likely reason for this is the lack of data from fundamental microvascular research. A better understanding of microvessel function will improve the criteria for assessing microcirculatory disorders, including point-of-care evaluation. Histohematic barriers perform a distinct function within the microcirculatory bed. They are formed by microvessel endothelial cells in close interaction with elements of the surrounding tissues: astrocytes in the central nervous system (forming the blood–brain barrier) and pericytes in other organs. The primary function of these barriers is to provide selective permeability, thereby shielding organ parenchymal cells from the direct influence of endogenous and exogenous pathogenic factors [20,21]. Given the above, the study of microvessels using histological and immunohistochemical methods seems particularly relevant. The central premise of the microvascular theory of pathology is the study of the cellular and molecular aspects of the interaction between microvessels and pathogens, as well as parenchymal cells, in a wide range of diseases—both infectious and non-infectious in nature.

## 2. Materials and Methods

We conducted a search in the Index Medicus database, followed by PubMed/MEDLINE, Scopus, and Web of Science, to identify literature relevant to the current topic. The evidence base for the new perspective was supported by the results of our own research published from 1989 to the present, as well as materials from a doctoral dissertation (1995). Data from a retrospective analysis and figures from histological and immunohistochemical studies are presented, covering both infectious and non-infectious diseases, as well as herpes (Table A1) and cryptococcal (Table A2) infections in laboratory animal experiments. Separately, literature data on non-infectious pathology were reviewed: diabetes mellitus, neurodegenerative diseases, atherosclerosis, and coronary heart disease. It is important to note that the results of previously conducted studies are still relevant today.

Before starting the study, all protocols were approved by the Local Ethics Committee of the Federal State Budgetary Educational Institution of Higher Education Saint Petersburg State Pediatric Medical University (№32/09 from 8 November 2023; №31/07 from 18 October 2023; 42/03 from 6 September 2024).

## 3. Results

### 3.1. Pathogenesis of Infectious Diseases: From the Cytopathic Effect to the Microvascular Genesis

#### Herpetic Encephalitis

Herpes simplex virus (HSV) remains a leading cause of encephalitis, associated with high mortality and disability rates [22]. The mechanism behind the rapid development of extensive necrotic brain lesions in herpetic encephalitis, which has long remained unclear, is of particular scientific and clinical interest. In the cases we observed, child mortality occurred within just 1–2 days after symptom onset due to diffuse necrosis of brain tissue. When the disease duration extended to 30 days, necrotic foci spread not only to the cerebral cortex but also to the midbrain.

Notably, alongside pronounced neuronal destruction, significant astrocyte activation was observed, manifested by their proliferation, hypertrophy of cell bodies, and processes. Experimental animal studies and postmortem observations confirmed that the astrocytic response persists, being more pronounced in the later stages of the infectious process. Astrocytes, forming the blood–brain barrier (BBB) together with capillary endothelium, serve as a key link between the vascular system and neurons. Astrocytes function as macrophages, act as immunocompetent cells in the brain, and participate in cytokine secretion.

**Figure 1:**
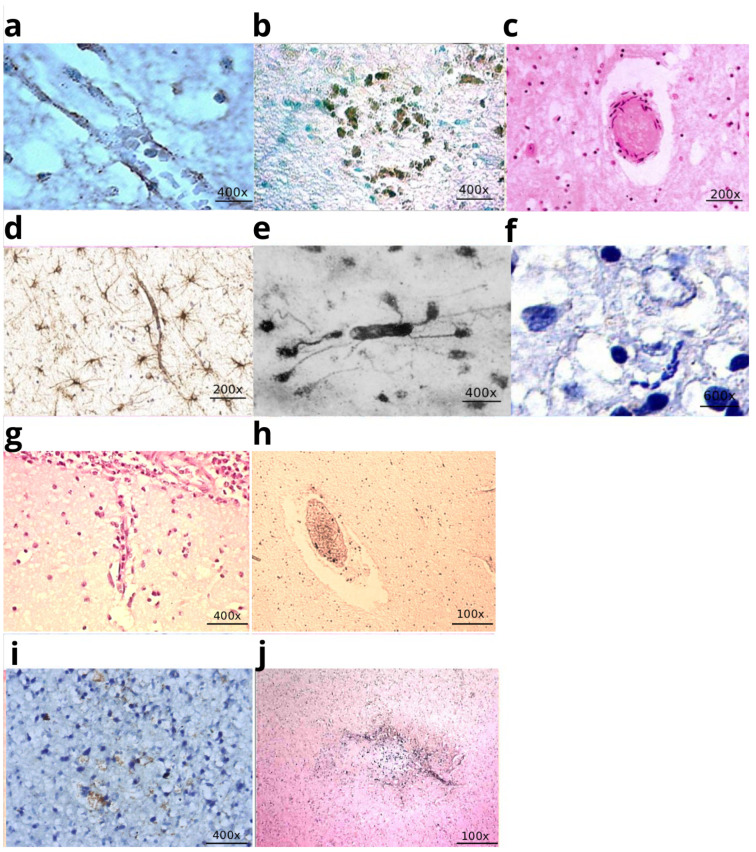
Microvessels in herpes infection and microvessels in infection caused by *Haemophilus influenzae*. (**a**) Herpes simplex virus antigen on the wall of a microvessel in a child who died of herpes encephalitis. IHC staining with Herpes simplex antibody; (**b**) Herpes simplex virus antigen on the wall of a microvessel and in the perivascular zone; (**c**) Mixed thrombus in the lumen of a microvessel. Infarction focus in the brain substance. H&E staining; (**d**) Perivascular reaction of astrocytes in the brain of a child who died of herpetic encephalitis, IHC staining with GFAP; (**e**) Perivascular proliferation of astrocytes, detachment of astrocytic vascular pedicles in the white matter of the hemispheres. Impregnation according to the Cajal method; (**f**) Cytoplasmic lysis, dark neurocyte nuclei in the cerebral cortex. Nissl stain with toluidine blue, ×600; (**g**) Disruption of the structural integrity of the arteriolar wall in the cerebral cortex of a child who died from a hemophilic infection. H&E staining; (**h**) Mixed thrombus in the lumen of a microvessel. Infarction in the white matter of the brain in a child who died from a hemophilic infection; (**i**) Expression of CD95 on the wall of microvessels in the cerebral cortex of a child who died from *Haemophilus influenzae* infection; (**j**) Expression of TNF on the wall of a microvessel and in the perivascular zone in a child who died from *Haemophilus influenzae* infection. The image was created based on the digital library on neuroinfection [30,31,32].

However, a paradox arises: if nerve cells are separated from the blood by the blood–brain barrier (astrocytes), how does the virus directly contact them? Nevertheless, the prevailing view explaining the cause of destructive damage to nerve cells remains the concept of the virus’s direct cytopathic effect on cells [23,24]. Viral replication is possible in the endothelium of cerebral vessels, where, according to the authors, mechanisms for bypassing the blood–brain barrier may be activated, followed by damage to neurons [25], which is theoretically difficult to imagine. At the same time, the viral antigen was selectively detected in the endothelium of microvessels (Figure 1a) and did not spread beyond the perivascular zone (Figure 1b). It was noteworthy that clusters of astrocytes were noted in this zone with swollen processes.

**Figure A2:**
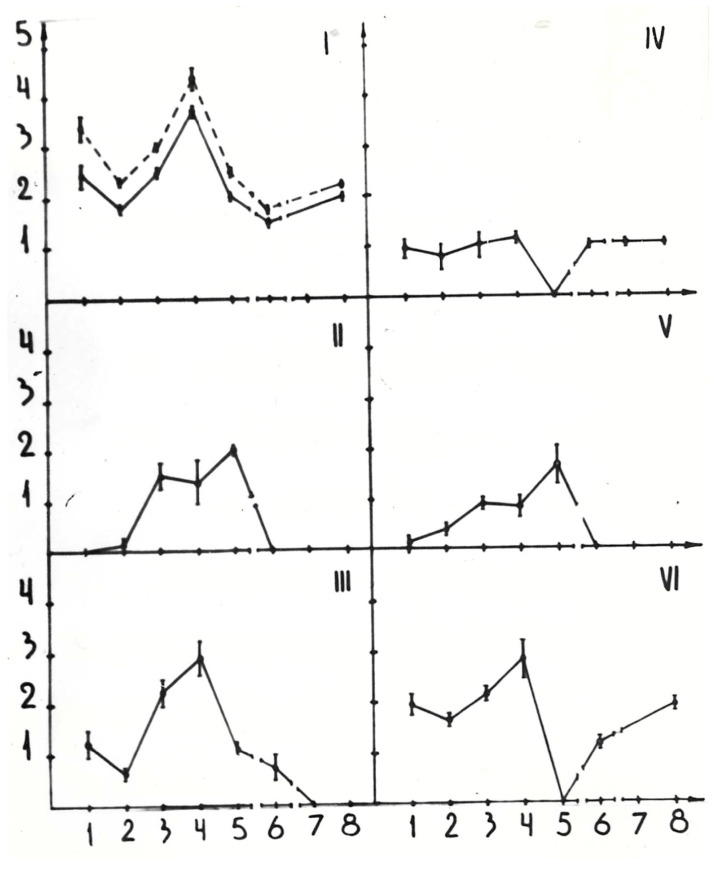
Dynamics of herpes simplex virus isolation from various organs and tissues of mice. The *x*-axis represents the days of the experiment; the *y*-axis represents virus activity (TCID 50). The solid line represents virus isolation by co-culturing organ cells with cells of a sensitive line; the dashed line represents virus isolation by hybridization of organ cells with cells of a sensitive line. I—brain; II—kidneys; III—blood; IV—lungs; V—spleen; VI—liver.

**Figure A3:**
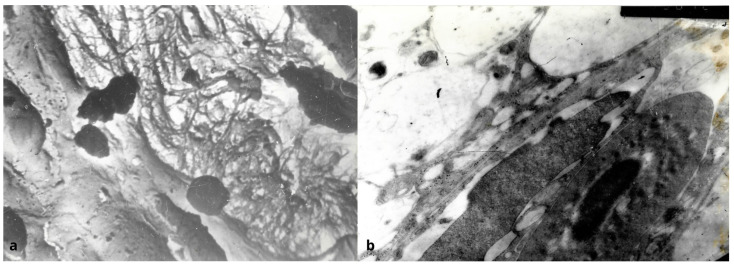
(**a**) Deendothelialization of the aortic zone. Fixation of blood cells on the 7th day of the experiment. Scanogram. Magnification ×4000; (**b**) Delamination, destruction of microvascular wall elements in the cerebral cortex on the 7th day of the experiment. Electron micrograph. Magnification ×10,000.

**Figure A4:**
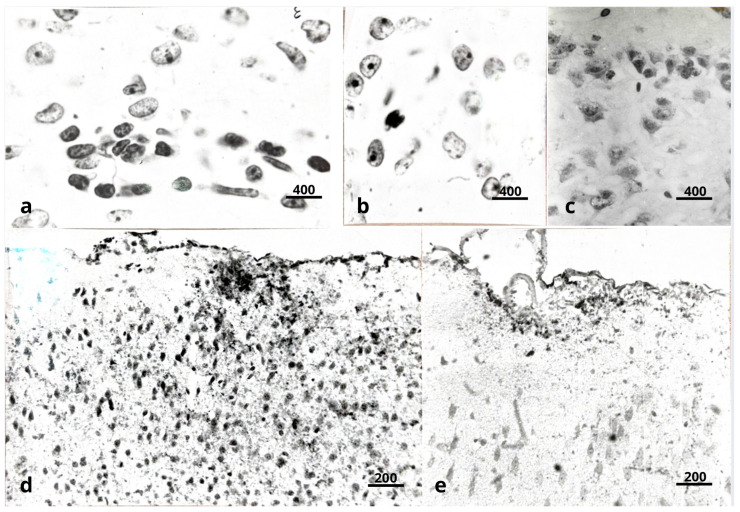
(**a**) Sharp expansion of the vessel lumen, change in the shape and size of endothelial cell nuclei. Swollen astrocyte nuclei. Day 3 of the experiment, Nissl staining, magnification ×400; (**b**) Increase in the number and size of astrocytes around microvessel fragments. Nissl staining, magnification ×400; (**c**) Hypoxic type of changes in nerve cells in the cerebral cortex of mice on day 7 of the experiment. Nissl staining, magnification ×400; (**d**) High intensity of expression of the herpes simplex virus antigen on the wall of microvessels and the perivascular zone in 1–2 layers of the cerebral cortex on day 3 of the experiment. IHC staining with antibodies to HSV and hematoxylin; magnification ×200; (**e**) Sharp decrease in the intensity of herpes simplex virus antigen expression on the wall of microvessels and the perivascular zone of the upper layers of the cortex and soft meninges on day 15 of the experiment. Hematoxylin staining, magnification ×200.

In children who died from herpetic encephalitis, severe lesions of the microvasculature were identified: deformation of the vascular lumen, foci of de-endothelialization, segmental necrosis of the walls, mixed thrombi (Figure 1c), and a combination of a proliferation reaction (Figure 1d) and detachment of astrocytic processes (Figure 1e). In neurons, Nissl staining revealed ischemic-type changes: hyperchromatic nuclei against a homogeneous cytoplasmic background, and ghost cells (Figure 1f). As shown in the illustration (Figure 1c) necrotic foci formed both near vessels and at a distance, merging into extensive areas of damage. These findings indicated an ischemic nature of the brain injury, which was also confirmed in experiments on mice (Figure A2, Figure A3 and Figure A4).

It is noteworthy that the literature almost entirely lacks studies linking necrotic lesions in herpetic encephalitis to vascular pathology. Only a few publications mention the similarity of histological changes in herpetic encephalitis to ischemic brain injuries [26], as well as the challenges in differential diagnosis between herpetic encephalitis and cerebral infarction [27].

The comprehensive analysis [28,29,30] conducted in this study provided the first substantiation for the concept of an ischemic genesis of necroses in herpes infection, which became a key postulate of the doctoral dissertation [31]. The data obtained served as the basis for further research and the development of the theory.

### 3.2. Expanding the Concept of Microvascular Genesis of Diseases: Haemophilus Influenzae Infection, Cryptococcosis of the Brain, Placental Examination, COVID-19

#### 3.2.1. Microvessels in Hemophilic Infection of the Brain

The rising incidence and mortality rates of *Haemophilus influenzae* meningitis [33] have underscored the need to investigate the morphogenesis of this cerebral infection. The mechanisms underlying brain tissue destruction in this bacterial neuroinfection remained unclear.

Histological and immunohistochemical (IHC) analyses, conducted jointly with M.V. Mankov, yielded new data explaining the cause of the severe brain damage. Thus, desquamation of endothelial cells and dissection of the microvessel walls were detected in the microcirculatory bed (MCB) of the pia mater and brain matter (Figure 1g). Mixed thrombi and fibrin deposition were detected in the lumens of capillaries and venules of the cortex and white matter. Foci of necrosis/infarction were detected near and distant from the affected microvessels (Figure 1h). A pronounced expression of the apoptosis marker CD95 was detected in the endothelium of cerebral capillaries, confirming endothelial cell death (Figure 1i).

Crucial for understanding the genesis of microvascular injury was the discovery of TNF-α protein molecule expression in the endothelium (Figure 1j) [32]. It is known that TNF-α induces apoptosis, affects endothelial cells, and modulates endothelial-leukocyte interactions, thereby promoting the disruption of intercellular junctions and morphological changes that lead to increased vascular permeability [34,35].

Conclusions. The molecular mechanism of endothelial cell death has been determined, as evidenced by the pronounced expression of the apoptosis marker CD 95 in the endothelium. The probable cause of apoptosis induction and microvascular wall damage is tumor necrosis factor-alpha (TNF-α), the expression of which has been detected in the endothelium. These changes—microvascular wall damage, thrombus formation, and the development of hypoxia—led to the formation of widespread foci of necrosis in the brain.

#### 3.2.2. Microvessels in Cryptococcal Infection of the Brain

**Figure 2:**
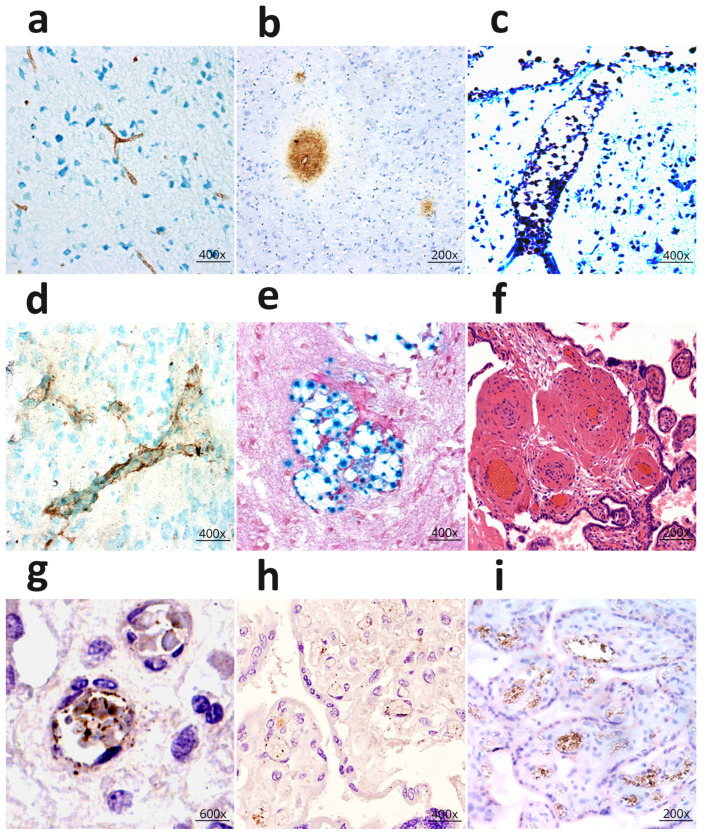
Microvessels of the brain in cryptococcal infection and placental damage. (**a**) Cryptococcus antigen (droplet polysaccharide) in the mouse brain. IHC; (**b**) Foci of pronounced expression of cryptococcal antigen in the perivascular zone of the mouse brain; (**c**) Cryptococci in the lumen of a microvessel, foci of necrosis in the cerebral cortex of a man who died of cryptococcosis. Stained with toluidine blue according to Nissl; (**d**) Pronounced expression of cryptococcal antigen on the wall of microvessels and perivascular zone in the brain of a woman who died from cryptococcal infection; (**e**) Large number of cryptococci in the area of a destroyed microvessel. Mowry staining; (**f**) Fibrin impregnation and necrosis of the capillary wall. Thrombi in the microvessels of the terminal villi of the placenta in the case of antenatal fetal death; (**g**) Expression of herpes simplex virus antigen in the endothelium of the capillaries of the placental villi; (**h**) Expression of CMV antigen in the endothelium of the microvessels of the intermediate villi; (**i**) TNF expression in the microvascular endothelium of intermediate villi in cases of antenatal fetal death). The image was created based on the digital library on neuroinfection [36,37,38].

Histopathological examination of the mouse brain revealed desquamation of the endothelium and homogenization of the walls in the cerebral microvessels as early as day 3 of the experiment, alongside hypoxic changes in the nerve cells. Thickening of perivascular astrocytic processes was also observed. Cryptococcal capsular antigen was detected in the microvascular endothelium and the perivascular zone of the brain parenchyma (Figure 2a).

During the acute phase of cerebral cryptococcosis, alongside intensified destructive changes in the microvessels, a progression of severe alterations in nerve cells and widespread foci of ischemic necrosis in the cortex were noted. An increased proliferative response of astrocytes, including in the perivascular zones, was observed. During this period, cryptococcal antigen was identified in the microvascular endothelium, perivascular spaces (Figure 2b), and within the cryptococcal capsules [36]. Neurohistological and immunohistochemical examination of the brains of individuals who died from cryptococcosis revealed a broad spectrum of changes comparable to and confirming the results from the experimental cryptococcosis model. Specifically, foci of coagulative necrosis were found, resulting from destructive changes in the walls of the cerebral microvessels. A large number of *cryptococci* were detected within the lumen of markedly dilated arterioles penetrating the cortex from the leptomeninges (Figure 2c). Cryptococcus antigen was detected on the wall of microvessels and in the perivascular zone (Figure 2d). Cryptococcus clusters were also detected at the sites of destroyed microvessels in the cortex and white matter (Figure 2e) [37].

Conclusions. Cryptococcal antigen was detected in the microvascular endothelium and the perivascular zone. Accumulations of cryptococci were identified at the sites of severely dilated and destroyed microvessels. Destructive changes in the microvasculature were the cause of ischemic necroses in cryptococcal neuroinfection. The data presented in this example of a fungal neuroinfection provide compelling evidence supporting the validity of the core principles of the microvascular genesis theory, thereby expanding our understanding of the development of the pathological process.

#### 3.2.3. Microvessels of the Placenta in Perinatal Mortality Cases

Perinatal mortality (PM), encompassing all fetal or newborn deaths from the 22nd week of pregnancy through the first 7 days after birth, remains a pressing issue in modern medicine. Despite extensive research, the causes of PM remain unidentified in over 70% of cases [39]. A number of studies suggest that herpes simplex virus (HSV) and cytomegalovirus (CMV) infections are responsible for spontaneous abortions, preterm births, and PM [40,41,42,43,44]. However, the mechanism of damage remained unclear. We conducted histological and immunohistochemical (IHC) studies of placentas from cases of antenatal and early neonatal death. Histological analysis revealed the presence of thrombi, significant alterations in capillary walls (Figure 2f), and structural changes in the placental terminal villi. In cases of antenatal and early neonatal death, increased expression of herpesvirus group antigens was detected. Specifically, marked expression of the HSV antigen (Figure 2g) and CMV antigen (Figure 2h) was observed in the capillary walls and the stroma of the placental villi. Notably, the localization of Tumor Necrosis Factor (TNF) molecules (Figure 2i) was found to coincide with that of the HSV and CMV antigens [38].

Conclusions. Based on our findings, the possibility of a direct cytopathic effect of the viruses on the cells was ruled out. Given the universal role of TNF and its capacity to induce apoptosis, we propose it as the primary cause of infected endothelial cell destruction and the development of thrombosis in the microvessels of the placental villi. The subsequent disruption of the placental-fetal circulation and the development of hypoxia are identified as the principal cause of antenatal and early neonatal mortality. The application of the microvascular concept enabled, for the first time, the elucidation of the sequence of pathological processes leading to perinatal death.

#### 3.2.4. Microvessels of the Lungs in COVID-19

**Figure 3:**
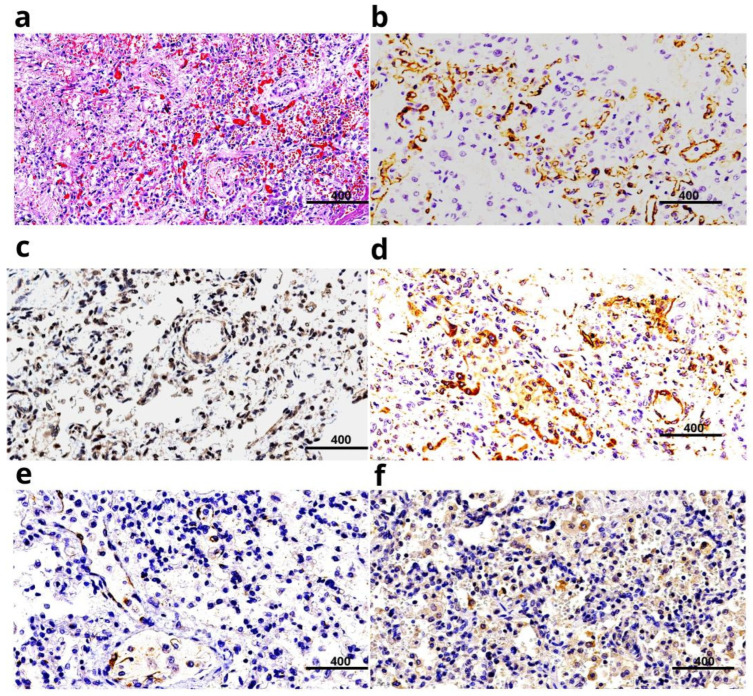
Microvessels of the lungs in COVID-19. (**a**) Thrombosis of the interalveolar septal capillaries and intra-alveolar hemorrhages were observed in a patient with COVID-19. H&E staining; (**b**) CD31 expression is observed in the endothelium of the microvasculature and the capillaries of the interalveolar septa. Discontinuity of the pulmonary capillary network is noted.; (**c**) Expression of SARS-CoV-2 nucleocapsid molecules on the wall of pulmonary microvessels; (**d**) Significant CD95 expression was detected in the endothelium of the microvasculature, the capillaries of the interalveolar septa, and in alveolar epithelial cells.; (**e**) IL-6 expression was detected in the endothelial cells of the microvasculature and in the capillary endothelium of the interalveolar septa in a patient with COVID-19 at 5–6 days after symptom onset; (**f**) IL-6 expression was observed in alveolar epithelial cells, alveolar macrophages, and the capillary endothelium of the interalveolar septa in a patient with COVID-19 at 20 days of disease duration. The image was created based on the digital library on COVID-19 [45,46].

We present the results of our own COVID-19 research as part of further investigation into the role of microvessels. We performed histological and IHC examinations of the lungs of four children who died from COVID-19 with predominant respiratory system involvement at different time points of the disease: 5, 21, 50, and 108 days. These data were interpreted considering clinical and laboratory parameters, as well as the results of ante- and post-mortem PCR testing for SARS-CoV-2. In all cases, vascular endothelial cell damage of various calibers was identified. Erythrocyte stasis, endothelial desquamation, and mixed clots were detected in the lumen of the capillaries of the interalveolar septa (Figure 3a). IHC analysis using antibodies of CD31 molecules demonstrated progressive destruction of the endothelial layer (Figure 3b). The most notable changes associated with an increase in the duration of COVID-19 were detected in the capillaries of the interalveolar septa. The investigation revealed high expression of the SARS-CoV-2 antigen in the endothelial cells of vessels (Figure 3c) of all sizes. Furthermore, marked expression of the apoptosis marker CD95 was observed in the alveolar epithelium, the endothelium of the interalveolar septa vessels (Figure 3d), and apoptotic bodies. It is also important that we identified increased expression of the apoptosis inducer molecule IL-6 in the microvascular endothelium, macrophages, and alveolar epithelium for the first time (Figure 3e,f) [45,46].

Conclusions. High expression of the SARS-CoV-2 nucleocapsid protein molecules was detected in the endothelial cells of vessels of all calibers and in the bronchial epithelium. The co-localization of the viral protein with CD95 and IL-6 molecules indicates the development of apoptosis in microvascular endothelial cells. Endothelial destruction, induced by apoptosis, was the cause of microvascular thrombosis. In turn, thrombosis led to tissue hypoxia and disseminated intravascular coagulation (DIC), ultimately resulting in multiple organ failure and a fatal outcome. Excluding the concept of a direct cytopathic effect of the virus on cells allowed us to propose a molecular mechanism for the pathogenesis of COVID-19. The obtained data could be used for the development of novel therapeutic agents for infectious diseases.

### 3.3. The Universality of the Microvascular Theory: Non-Infectious Diseases

#### 3.3.1. Microvessels of the Skin in Lichen Sclerosus

Lichen sclerosus (LS) is a chronic inflammatory disorder of the skin and mucous membranes, pathologically defined by epidermal atrophy and dermal sclerosis. Contemporary studies implicate autoimmune mechanisms and hormonal imbalances as key etiological factors [47,48]. The present study [49] identified significant microvascular alterations in female patients with LS that demonstrated interdependence with disease duration. In early-stage disease (≤1 year), histopathological observations included capillary and arteriole proliferation, endothelial cell edema, and luminal thrombosis. Immunohistochemical analysis at this stage revealed preserved CD31 expression, albeit with discontinuous staining in 30% of vessels, indicative of incipient endothelial damage. A marked upregulation of VEGF in 80% of vessels was noted, potentially reflecting a compensatory angiogenic response to localized hypoxia, while weak CD95 expression in 20% of cells signified the initial activation of apoptotic pathways.

**Figure 4:**
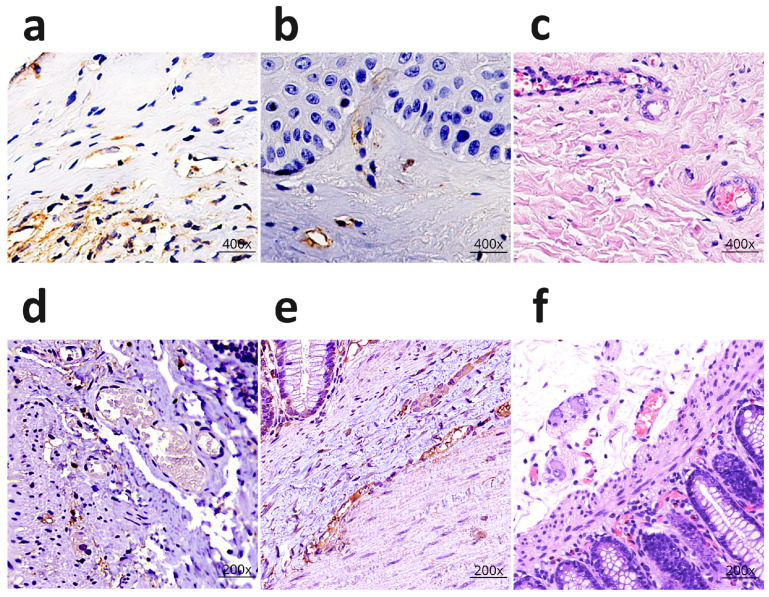
Microvessels in lichen sclerosus and microvessels in Hirschsprung’s disease. (**a**) Intensive expression of the CD 95 in the endothelium of the microvessels, inflammatory infiltrate cells in lichen sclerosus, ×400; (**b**) Expression of the CD 31 in the microvessels in lichen sclerosus, ×400; (**c**) Mixed clot in a microcirculatory vessel. Dermal sclerosis in lichen sclerosus. H&E staining, ×400; (**d**) Expression of CD95 in the capillary wall of the intestinal mucosa, ×200; (**e**) Expression of IL-6 in the capillary walls of the intestinal mucosa and neurons of the submucosal ganglia, ×200; (**f**) Mixed thrombi in microvessels in the submucosal plexus. H&E staining, ×200. The image is based on the digital library on non-infectious diseases [49,50,51].

In contrast, late-stage disease (≥2 years) was characterized by profound endothelial destruction, evidenced by nuclear fragmentation, ballooning degeneration, and fibrin deposition within the microvessel walls. Immunohistochemical profiling confirmed massive apoptosis, with CD95 expression (Figure 4a) markedly elevated in 95% of cells. Concurrently, CD31 expression was substantially reduced and uneven in vessels (Figure 4b) corroborating the extensive loss of endothelial integrity. VEGF expression was sharply diminished, present in only 15% of vessels, consistent with a depletion of angiogenic capacity. The presence of mixed thrombi within the microvasculature and subsequent sclerotic changes (Figure 4c) in the perivascular tissue are posited as direct consequences of persistent dermal hypoxia [50].

Conclusions: The results indicate that the primary target of the pathogenic agent (likely autoantibodies, toxic metabolites, etc.) is the microvascular endothelium of the skin. It was established that the disruption of the endothelial structural integrity, caused by cell apoptosis, leads to impaired microcirculation, increased vascular permeability, and the formation of mixed thrombi. The ensuing tissue hypoxia resulting from these processes underlies the key manifestations of LS: the development of atrophic foci in the epidermis, and inflammatory infiltration and sclerosis in the dermis.

#### 3.3.2. Microvessels of the Rectum in Hirschsprung’s Disease

This study analyzed the results of histological and IHC examinations of surgical specimens from the rectal segments of 10 children. Histological examination revealed characteristic changes in the microvasculature. In arterioles, the walls showed fiber dissociation and edema, with swollen endothelial cell nuclei. The lumen contained aggregates of erythrocytes and fibrin strands. In capillaries, the endothelial cell nuclei were shrunken and flattened. The most pronounced alterations were found in venules. The lumen acquired a stellate or slit-like appearance; the walls exhibited fibrin impregnation, thickening, and homogenization. In isolated cases, mixed thrombi were identified. Immunohistochemical staining for GFAP demonstrated an increased number of glial cells surrounding the damaged neurons (up to 20 per field of view), which may indicate a neuroprotective or neurodegenerative response. Toluidine blue Nissl staining revealed hypoxic changes in the neurons, including nuclear hyperchromatosis and homogenization of the chromatophilic substance. In severe cases, expression of the apoptosis marker CD95 and reduced expression of calretinin were observed, indicating a disruption of the structural integrity of the enteric nervous system. IHC analysis did not show significant CD95 expression in the endothelium (Figure 4d). However, pronounced expression of IL-6 molecules in endothelial cells (Figure 4e) may suggest the activation of an alternative apoptotic pathway [52]. The nature of the histological changes also demonstrates severe alterations in the endothelial cells. In one quarter of the cases, examination revealed the presence of mixed thrombi (Figure 4f) [51].

Conclusions: The study identified a complex of pathological changes in the rectal microvasculature (arterioles, capillaries, and venules), including apoptosis of endothelial cells, fibrinoid wall impregnation, and thrombosis. Damage to the microvascular endothelium leads to the development of hypoxia, as evidenced by the activation of glial cells and neuronal loss (expression of CD95 molecules, decreased calretinin expression). The identified sequence of alterations in the rectal wall provides a new approach to understanding Hirschsprung’s disease within the framework of the proposed microvascular theory of pathology.

#### 3.3.3. Microvessels in Diabetes Mellitus

As is known, vascular complications in diabetes mellitus (DM) represent a leading cause of morbidity and mortality among this patient cohort. Their development is driven by a complex interaction of systemic metabolic disturbances (hyperglycemia, dyslipidemia) and local tissue responses to cytotoxic metabolites. Microvascular complications of DM traditionally include retinopathy, nephropathy, and neuropathy. However, the pathological process also affects the brain, myocardium, skin, and other organs. In both type 1 and type 2 DM, progressive damage to the microvasculature is observed, leading to tissue hypoxia and determining the severity of the disease [53].

According to current data [54], endothelial cells possess a limited capacity to regulate glucose transport under conditions of chronic hyperglycemia, rendering them particularly vulnerable to intracellular glucose accumulation and subsequent oxidative stress. A key pathogenetic factor in the development of diabetes mellitus, according to [55], is hypercoagulability and thrombus formation in the microvessels, which induces tissue ischemia.

Conclusions: (1) The presented data reflect the structural and functional alterations in the microvasculature that underpin the pathogenesis of diabetes mellitus. (2) Diabetic microangiopathy is a multifactorial process affecting various target organs. Understanding its molecular foundations opens prospects for developing targeted therapeutic strategies aimed at correcting endothelial dysfunction and preventing the progression of DM complications.

#### 3.3.4. Microvessels in Neurodegenerative Diseases

The critical importance of cerebral blood supply for brain function is universally acknowledged [56]. Nevertheless, neuroscience long adhered to an approach that considered neurons and the cerebrovascular system in isolation. Similarly, a strict nosological distinction was maintained between classical neurodegenerative diseases, such as Alzheimer’s disease, and cerebrovascular pathology, such as stroke. A fundamental shift in understanding the interaction between neurons and vessels occurred following the formulation of the neurovascular unit (NVU) concept in 2001 “https://www.ninds.nih.gov/About-NINDS/Strategic-Plans-Evaluations/Strategic-Plans/Stroke-Progress-Review-Group (accessed on 26 November 2025), which emphasized their symbiotic relationship. According to this concept, the functional integration of vessels, glia, and neurons plays a key role not only in maintaining cerebral homeostasis but also in the pathogenesis of brain disorders [57]. Accumulated evidence indicates that chronic cerebral hypoperfusion may play a central role in the development of Alzheimer’s disease, preceding the onset of cognitive impairment and the accumulation of β-amyloid [58]. Research confirms that the state of the microvasculature critically influences the pathogenesis of Alzheimer’s disease, acting both as a target and as a factor exacerbating neurodegenerative processes [56]. Structural and functional impairments in neuronal networks, associated with microvascular damage and dysregulation of the blood–brain barrier, are observed in various neurodegenerative diseases, including Alzheimer’s disease, frontotemporal dementia, amyotrophic lateral sclerosis, idiopathic Parkinson’s disease, and dementia with Lewy bodies [57]. It is noteworthy that as early as the 1970s–1980s, neuromorphology had already developed the concept of the “vessel-glia-neuron” system, reflecting their structural and functional interdependence. This once again confirms the relevance of the approach based on anatomical and histological studies (Virchow) in investigating disease pathogenesis.

Conclusions: (1) Current evidence indicates that neurodegenerative diseases represent pathological processes mediated by microvascular dysfunction. (2) Undoubtedly, further investigation of the microvascular mechanisms of neurodegeneration opens new prospects for developing diagnostic and therapeutic strategies for this group of severe and increasingly prevalent diseases.

#### 3.3.5. Microvessels in Ischemic Heart Disease

This review [59] provides a detailed analysis of data highlighting the pivotal role of coronary microvascular dysfunction (CMD) in the pathogenesis of ischemic heart disease (IHD). The primary pathogenetic mechanism of CMD involves structural and functional remodeling of the microcirculatory network (arterioles < 100 μm in diameter and capillaries), leading to impaired autoregulation of coronary blood flow [60].

The clinical significance of CMD is evident across various forms of IHD:

In acute myocardial infarction, CMD serves as an independent predictor of heart failure and mortality [61,62,63]. In chronic IHD, it exacerbates myocardial ischemia [64]. In ischemia with no obstructive coronary arteries (INOCA), CMD is the primary cause of recurrent angina [65,66]. In hypertrophic cardiomyopathy, it is an independent predictor of mortality [67].

**Figure 5:**
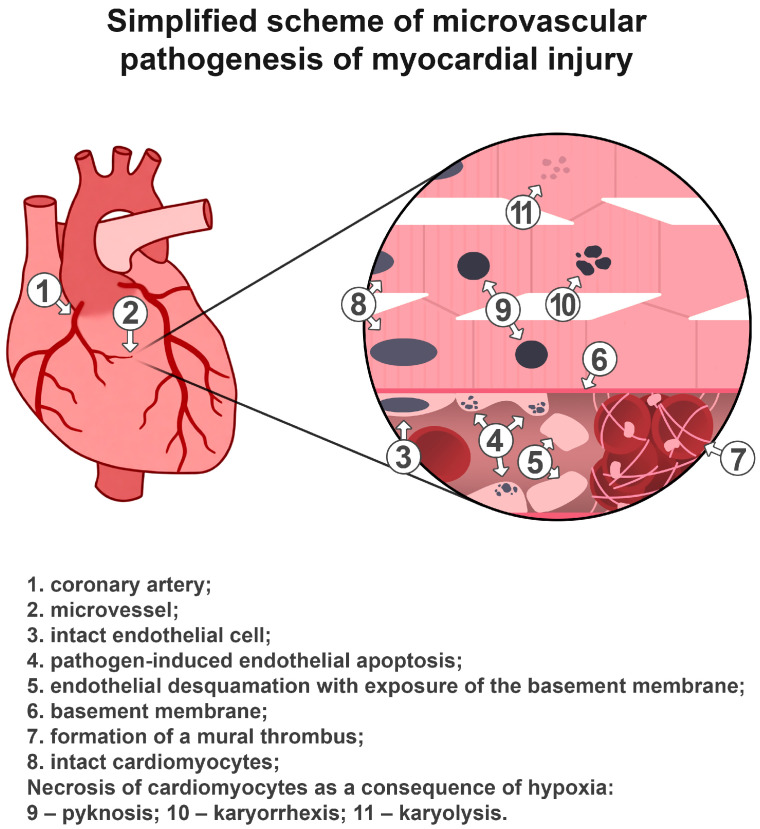
The sequence of development of the myocardial lesion.

**Figure A5:**
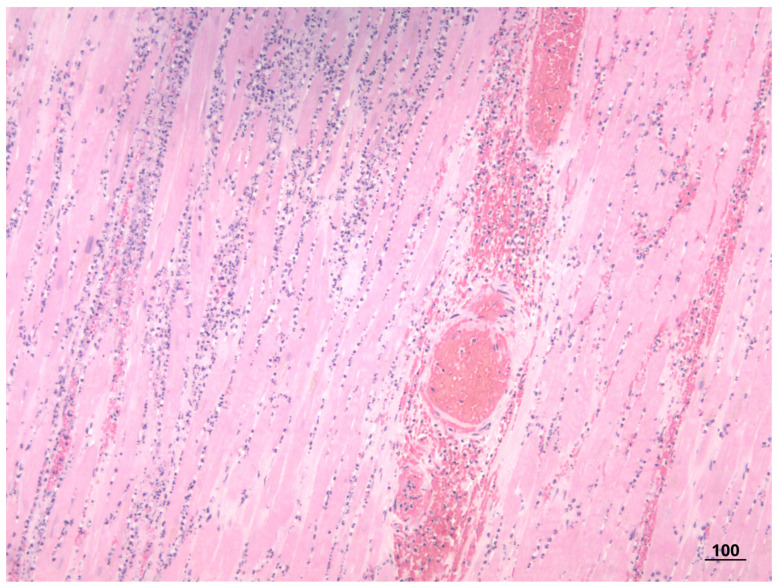
Myocardial alterations in a patient with myocardial infarction. Thrombosis of the microcirculatory bed vessels. Extensive perivascular hemorrhage, desquamation of endotheliocytes. H&E staining.

The sequence of development of the myocardial lesion is shown in Figure 5, and the nature of morphological changes in the lesion is shown in Figure A5.

## 4. Discussion

This study examined a wide spectrum of infectious and non-infectious diseases. The application of selective histological staining techniques enabled a detailed investigation of alterations within the microcirculatory system, including the vascular wall and perivascular tissues across various organs. IHC analysis not only corroborated the histological findings but also elucidated molecular aspects of the pathogen and macroorganism interaction.

In the early stages of infectious processes, pathogen antigens were localized to the microvascular endothelium. A subsequent, key argument underscoring the pivotal role of the vascular component is the correlation between TNF expression and the development of endothelial cell apoptosis. In cases of COVID-19 and Hirschsprung’s disease, interleukin-6 (IL-6) acted as the inducer of apoptosis within the microvasculature of the lungs and rectum, respectively. We have previously emphasized that we do not consider the direct cytopathic effect of viruses on endothelial cells. We view them as sites of viral replication, not as targets of their cytopathic effect [38,45]. COVID-19-associated endothelial dysfunction is a pivotal pathogenetic factor driving disease severity and complications. Pro-inflammatory cytokine-mediated endothelial damage (e.g., by IL-6 and TNF) leads to tissue factor expression, suppression of anticoagulant activity, and von Willebrand factor release [68]. This promotes thrombogenesis in vessels of varying caliber [69]. Research demonstrates that biochemical markers of endothelial dysfunction, including elevated von Willebrand factor and soluble thrombomodulin, along with microthrombosis, persist in Long COVID patients up to one year following mild acute infection [70]. This results in microcirculatory dysfunction and reduced tissue oxygenation. Thus, these findings potentially explain commonly reported symptoms: chronic fatigue, cognitive dysfunction, and exercise intolerance [71]. Similarly, some authors argue that COVID-19-associated endothelial dysfunction may be considered a long-term predictor of early atherosclerosis progression [72].

Another aspect is noteworthy, which may also change our understanding of the infectious process. Histological analysis demonstrated that *Cryptococcus* persists within the lumen of microvessels even during the late stages of infection. By this time, the vascular architecture is significantly disrupted, with only fragments of microvessels identifiable at the periphery of cryptococcal accumulations. Cryptococcal antigen was detected on the microvascular wall and in the perivascular zone. This suggests that cryptococcal aggregates do not leave the vascular lumen, and individual cryptococci do not penetrate beyond the perivascular zone of the brain parenchyma. This observation is clearly visualized histologically and confirmed by IHC data. A similar conclusion is drawn regarding herpes infection. IHC findings indicated that herpes simplex virus antigen was not detected beyond the perivascular zone, pointing to selective targeting of the microcirculatory system. Despite the importance of these results, these remain preliminary conclusions. Nevertheless, the notion that pathogens do not cross the blood–brain barrier aligns with the principles of the microvascular theory of pathology. This may refer to the biological rationale behind the behavior of pathogenic microorganisms, aimed at maximizing their survival, reproduction, and dissemination within the host population. The mechanism by which infectious agents, particularly *Cryptococcus*, traverse the blood–brain barrier remains a challenging question in medicine [73,74]. The protective reaction of astrocytes around microvessels, identified in our study, likely plays a significant role in explaining why pathogens do not advance beyond the perivascular zone.

Understanding the interaction of damaging agents with the blood–brain and histohematic barriers is fundamental for elucidating pathogenesis and developing novel treatments for both infectious and non-infectious diseases. In all cases studied, damage to the microvasculature, including components of the blood–brain and histohematic barriers, was accompanied by hypoxic changes in parenchymal cells, culminating in the formation of extensive necrotic foci. These observations suggest a common pathogenetic mechanism for infectious agents of diverse nature.

In the case of the non-infectious disease Lichen Sclerosus, histological and immunohistochemical studies demonstrated that the key pathogenic element is also damage to the microvascular wall. For the first time, an interrelationship has been established between structural changes in the “vessel-glia-neuron” system within the rectal wall in Hirschsprung’s disease. It was discovered that primary microvascular damage precedes degenerative changes in neuronal elements, challenging traditional views on the pathogenesis of this disease.

Translating histological findings into clinical biomarkers is a relevant interdisciplinary challenge. This task involves standardizing morphological features and converting them into quantitative parameters. This is accomplished through standardized scoring systems that convert qualitative observations into diagnostic and prognostic markers. The implementation of these protocols, alongside routine methods, increasingly involves the application of machine learning and neural network techniques [75,76,77].

We employed conventional visualization methods in our study. Thus, the combination of quantitative and qualitative microvascular parameters, along with the expression of CD31 (an endothelial marker), VEGF (a vascular growth marker), and CD95 (an apoptosis marker), is transformed into a clinical biomarker for staging and the differential diagnosis of Lichen Sclerosus, as well as other examples from the group of non-infectious diseases. The quantitative reduction in neuronal population and the atrophy of neuronal cell bodies are translated into a clinical biomarker that reflects the aggravation of Hirschsprung’s disease course in the pediatric population. An increased glial cell count (≥20 per neuron) serves as a clinical biomarker for neuronal pathology. The combination of weak CD95 and strong IL-6 expression serves as a clinical biomarker for aggravated manifestations of Hirschsprung’s disease. The use of artificial intelligence for further results processing becomes feasible as the database expands. It should be noted that the novel understanding of disease pathogenesis broadens the potential for diagnosis, prognosis, and targeted therapy.

The conducted research indicates that damage to parenchymal cells in both infectious and non-infectious diseases is a secondary phenomenon. Furthermore, it should be added that the effects of administered drugs, as well as endogenous factors, are also mediated by alterations in the microvasculature. The entire interaction process is multi-stage, and a profound understanding of it has the potential to initiate significant progress in medical science and practice. This is supported, in particular, by clinical observational data highlighting the role of microcirculatory disturbances in severe non-infectious pathologies. It has been established that endothelial dysfunction is a key trigger in the pathological process of diabetes mellitus, leading to increased vascular permeability [78], thrombosis, and tissue ischemia [55]. Undoubtedly, understanding the molecular basis of diabetes pathogenesis opens prospects for developing targeted therapeutic strategies aimed at correcting endothelial dysfunction.

The evolution of modern concepts regarding the pathogenesis of neurodegenerative diseases has become an important argument in favor of the microvascular theory of pathology. The established neurovascular unit concept emphasizes the interdependence of cerebral vessels, glia, and neurons. Research confirms that the state of the microcirculation can act both as an early target and as a factor exacerbating neurodegeneration [56], which is entirely consistent with the principles of the proposed microvascular theory.

Increasing attention is currently being paid to the study of microvascular pathology within the arterial wall (vasa vasorum), opening new perspectives for understanding the mechanisms of atherosclerosis development [79,80], and possibly other diseases. Data from the literature review presented above indicate that coronary microvascular dysfunction is an integral component of the pathogenesis of ischemic heart disease, capable of inducing myocardial ischemia even in the absence of epicardial artery obstruction. Its role is confirmed in acute and chronic forms of IHD, as well as in genetically determined cardiomyopathy. The increasing number of randomized controlled trials aimed at elucidating specific treatments for coronary microvascular dysfunction, noted in several reviews [81,82,83], may indicate a growing recognition of microvascular pathology as a dominant factor in the development of IHD. The development of personalized therapies for microvascular damage in other diseases can also be anticipated. It should be noted that the development of hypoxia, as a factor of damage in various diseases, can also be considered as an aspect for new therapeutic treatment strategies [84].

An important direction in treatment appears to be the application of antithrombotic agents. This conclusion is based on the results obtained in our original studies. We found that both in infectious and non-infectious diseases, TNF and IL-6 play a key role in inducing apoptosis in the microvascular endothelium. This finding provides a rationale for the earlier administration of anticytokine therapy to halt the pathological process. Currently, the scope of research targeting TNF and IL-6 is expanding. For instance, researchers have proposed the use of Diquafosol for the treatment of dry eye syndrome [85], statin therapy for kidney diseases [86], and dietary interventions for obesity [87]. Furthermore, the authors demonstrated the superiority of a bispecific nanobody targeting both TNF and IL-6 simultaneously [88]. However, several studies have emphasized that the administration of anticytokine therapy alone (for example, with Tocilizumab) is insufficient. Thus, Kovács E.H. et al. in their article demonstrate that while a reduction in inflammation was observed following the administration of anti-IL-6 drugs, COVID-19-associated coagulopathy persisted [89].

In conclusion, the presented body of work encompasses a broad range of investigations, from the molecular mechanisms of endothelial injury and microthrombosis to the clinical manifestations of microcirculatory disturbances in infectious and non-infectious diseases. These studies underscore the pivotal role of microcirculatory dysfunction in the development of organ failure, including multi-organ failure.

## 5. Conclusions

1. The microvasculature, including barrier structures, is the primary target for various pathogens; their damage serves as the trigger for the pathological process in organs.

2. The progression of the pathological process, involving microvascular wall damage, increased permeability, and thrombus formation, leads to hypoxia. This hypoxia determines the nature of the changes in the surrounding tissue, up to the formation of necrotic foci (infarcts).

3. The interaction between the pathogen and the host organism exhibits two distinct phases: phase 1—from the pathogen’s entry into the bloodstream to its impact on the microvascular structures; phase 2—the consequences of microvascular damage in the parenchymal cells of the organs.

4. The identified stereotypical pattern of changes in the microvasculature and surrounding tissues confirms the commonality of the pathogenesis of infectious and non-infectious diseases and will allow the use and creation of drugs to target the main links in the pathological process: wider use of antithrombotic, endothelium-protective, anti-cytokine agents, etc.

5. Acceptance of the microvascular theory instead of traditional models changes our understanding of pathology, opening up new directions for research into the pathogenesis of diseases and the development of personalized therapeutic methods.

6. The proposed Microvascular theory of pathology represents a modern and evidence-based approach to understanding disease pathogenesis. It is grounded in the theories of K. Rokitansky and R. Virchow, integrating fundamental and clinical aspects of medicine.

## Data Availability

The original contributions presented in the study are included in the article, further inquiries can be directed to the corresponding author.

## Abbreviations

The following abbreviations are used in this manuscript:

MCR	Microcirculatory system
BBB	Blood–brain barrier
HSV	Herpes simplex virus
IHC	Immunohistochemical
GFAP	Glial Fibrillary Acidic Protein
TNF	Tumor Necrosis Factor
MCB	Microcirculatory bed
CMV	Cytomegalovirus
PM	Perinatal mortality
PCR	Polymerase Chain Reaction
IL-6	Interleukin-6
DIC	Disseminated intravascular coagulation
LS	Lichen sclerosus
VEGF	Vascular Endothelial Growth Factor
DM	Diabetes mellitus
NVU	Neurovascular unit
CMD	Coronary microvascular dysfunction
IHD	Ischemic heart disease
INOCA	Ischemia with no obstructive coronary arteries
TCD	Tissue Culture Dose

## Appendix A

**Table A1:** Experiment with HSV infection.

Group	Pathogen, Infective Dose	Mode of Infection
Control group of mice (*n* = 10)	-	-
Experimental group of mice (*n* = 50)	Herpes simplex virus type 1 (HSV-1) strain L2, 100 TCD50/0.02.	Intracerebral inoculation

**Table A2:** Experiment with Cryptococcus infection.

Group	Pathogen, Infective Dose	Mode of Infection
Control group of mice (*n* = 10)		
Experimental group of mice (*n* = 50)	Cryptococcus neoformans strain 1178, dose of 106 cells per mouse.	Injection into the tail vein
